# Cellular RelB interacts with the transactivator Tat and enhance HIV-1 expression

**DOI:** 10.1186/s12977-018-0447-9

**Published:** 2018-09-21

**Authors:** Meng Wang, Wei Yang, Yu Chen, Jian Wang, Juan Tan, Wentao Qiao

**Affiliations:** Key Laboratory of Molecular Microbiology and Technology, Ministry of Education, College of Life Sciences, Nankai University, Tianjin, 300071 China

**Keywords:** LTR, Tat, RelB, Transcription

## Abstract

**Background:**

Human immunodeficiency virus type 1 (HIV-1) Tat protein plays an essential role in HIV-1 gene transcription. Tat transactivates HIV-1 long terminal repeat (LTR)-directed gene expression through direct interactions with the transactivation-responsive region (TAR) element and other *cis* elements in the LTR. The TAR-independent Tat-mediated LTR transactivation is modulated by several host factors, but the mechanism is not fully understood.

**Results:**

Here, we report that Tat interacts with the Rel homology domain of RelB through its core region. Furthermore, RelB significantly increases Tat-mediated transcription of the HIV-1 LTR and viral gene expression, which is independent of the TAR. Both Tat and RelB are recruited to the HIV-1 promoter, of which RelB facilitates the recruitment of Tat to the viral LTR. The NF-κB elements are key to the accumulation of Tat and RelB on the LTR. Knockout of RelB reduces the accumulation of RNA polymerase II on the LTR, and decreases HIV-1 gene transcription. Together, our data suggest that RelB contributes to HIV-1 transactivation.

**Conclusions:**

Our results demonstrate that RelB interacts with Tat and enhances TAR-independent activation of HIV-1 LTR promoter, which adds new insights into the multi-layered mechanisms of Tat in regulating the gene expression of HIV-1.

**Electronic supplementary material:**

The online version of this article (10.1186/s12977-018-0447-9) contains supplementary material, which is available to authorized users.

## Background

Human immunodeficiency virus type 1 (HIV-1) encodes a transcriptional activator protein Tat, which regulates multiple stages of HIV-1 transcription, robustly increases viral RNA synthesis, and allows efficient viral replication [1–3]. Transcription of integrated proviral HIV-1 DNA is initiated from the long terminal repeat (LTR). The LTR is a strong RNA polymerase II (RNA Pol II) promoter that contains many binding sites for cellular transcription factors [3–5]. Tat potently transactivates HIV-1 LTR-directed gene expression by directly interacting with the nascent RNA stem-loop known as the transactivation-responsive region (TAR) and promoting transcription elongation [6]. In addition to TAR, several *cis*-acting regulatory elements in the HIV-1 LTR are also essential for Tat activation. These elements include the proximal enhancer element containing two NF-κB binding motifs, and the core element containing three Sp1-binding sites, a TATA element, and an initiator element [3, 7–11]. Das et al. generated an HIV-1 proviral DNA construct that does not depend on the Tat-TAR interaction for transcription and demonstrated that Tat remains important for viral transcription via the Sp1-binding sites in the U3 region of LTR promoter [12, 13]. It is therefore posited that Tat is able to promote viral replication at the very early stage of HIV-1 transcription before TAR is formed [14]. Multiple host cellular factors such as Sp1, nuclear factor κB (NF-κB), TATA-binding protein (TBP), nuclear factor of activated T cells (NFAT), are essential for the TAR-independent transcriptional activity of Tat [7, 9, 15, 16].

NF-κB binding sites are found in the enhancer region of all primate lentiviral LTRs, although the number may vary between different subtypes of HIV-1. Most subtypes of pandemic HIV-1 group M strains (A, B, D, F, G, H, J, and K) contain two NF-κB binding sites located − 104 to − 80 bp upstream of the transcription start site within the 5′ LTR. In contrast, human immunodeficiency virus type 2 (HIV-2) and the A/E recombinant of HIV-1 contain a single NF-κB binding site. Subtype C strains typically contain three binding sites of NF-κB in their enhancer regions [17, 18]. It is known that HIV-1 transcription can be enhanced by activation of the NF-κB pathway [19, 20]. Earlier studies have shown that Tat can directly bind to the NF-κB binding sites [21, 22] and activate NF-κB via physical interactions with IκBα and RelA at the initiation step of transcription [23].

To further illuminate cellular co-factors that assist the role of Tat in transcription initiation, we screened for Tat-binding cellular protein using a human B cell library in a yeast two-hybrid assay, and RelB was one of those proteins that were identified. RelB is a member of the NF-κB family of transcription factors (p50/p105, p52/p100, RelA, RelB and c-Rel) [24, 25]. It is structurally and functionally different from the other two transcriptionally active members, RelA and c-Rel [26]. In addition to a Rel homology domain (RHD) and a transactivation domain (TAD), RelB also has an N-terminal leucine zipper domain that is not present in other NF-κB family members [24]. RelB binds to the target promoters through interaction with other proteins (e.g. p52, p50 and RelA). Compared to other members of the NF-κB family, the mechanism of action of the RelB subunit has remained elusive [27, 28]. Moreover, several viral components, such as HIV-1 Vpr, bovine foamy virus (BFV) transactivator BTas, and human T cell leukemia virus type 1 (HTLV-1) Tax1, have been demonstrated to interact with RelB to facilitate virus replication or contribute to the pathogenesis of the disease [29–31]. However, in these studies, the role of interaction between Tat and RelB in activating the LTR promoter was not investigated.

Our study shows that RelB interacts with Tat, and they are recruited to the HIV-1 promoter. The NF-κB elements are key to the association of Tat and RelB with the LTR. RelB regulates proviral DNA transcription by enhancing the effect of Tat in activating LTR transcription independent of TAR. Thus, we have identified RelB interacts with Tat and enhances the TAR-independent activation of HIV-1 gene expression.

## Methods

### Plasmids

DNA constructs pCMV-Tat [32], pFlag-Tat, pMyc-Tat, pEGFP-Tat [33], pCMV-RelB, pFlag-RelB, pMyc-RelB [29], pCMV-p100 [34], pHIV-1-LTR-luc [30, 32], pQCXIP (pRetroX-Tight-Pur retroviral vector, Clontech), pMLV-Gag-Pol and pVSV-G were described previously [35]. HIV-1 proviral DNA clone pNL4-3.Luc.env^−^ was a gift from Johnny J He [36]. This proviral DNA construct was derived from the NL4-3 strain with env gene inactivated and the firefly luciferase gene in place of HIV-1 nef [35]. The pNLENY1-ES-IRES construct was kindly provided by Dr. David Levy (University of Alabama at Birmingham, USA) [37]. Plasmids pLentiCRISPR (pXPR_001) and psPAX2 (AddGene 12260) were kindly provided by Dr. Feng Zhang (MIT, USA) [38]. Plasmids pHA-RelB, and pQC-RelB were constructed by PCR amplification of pCMV-RelB and cloning of PCR products into pCMV-3HA, or pQCXIP vectors. pHA-Tat was constructed by inserting Tat with a C-terminal HA tag into the pQCXIP retroviral vector. RelB deletion mutants were described previously [29]. Tat deletion mutants were engineered by inserting the respective PCR fragment into pEGFP-C1. Constructs of pHIV-1-LTR-luc deletions were generated through PCR using pHIV-1-LTR-luc as a template and cloned into pGL3-basic (promega, CAT# E1751), that were described previously [32]. Plasmids pHIV-1-LTR-mutTAR, pHIV-1-LTR-mutNFκB, and pHIV-1-LTR-mutTAR-mutNFκB were generated using PCR-based mutagenesis. The PCR primers are listed in Additional file 1: Table 1. The sequences of all new constructs were confirmed by sequencing.

### Cell culture and transfection

HEK 293T (ATCC, CRL-11268G-1), HeLa (ATCC, CCL-2), and TZM-bl cells (NIH, CAT# 8129) [39–41] were maintained in Dulbecco’s modified Eagle’s medium (DMEM, Gibco) with 10% fetal bovine serum (FBS, Hyclone). Jurkat (ATCC, TIB-152), J-Lat A72 (NIH, CAT# 9856) [42, 43], and peripheral blood mononuclear cells (PBMCs) were maintained in RPMI 1640 (Corning) with 10% FBS and 2.4 mM l-glutamine (Gibco). Cells were maintained in a humidified atmosphere containing 5% CO_2_ at 37 °C. HeLa, TZM-bl, HEK 293T cells were transfected by polyethylenimine (PEI, Poly-sciences, CAT# 23966) or Lipofectamine 2000 (Invitrogen, CAT# 11668-019) in accordance with the manufacturer’s instructions.

PBMCs were isolated from buffy coat from healthy blood donors by histopaque and percoll gradient centrifugation, and were activated with phytohemagglutinin (PHA, 5 μg/ml) (Sigma, CAT# L1668) and IL-2 (20 U/ml) (Pepro Tech, CAT# 200-02) for 24 h before virus infection [35]. The siRNAs (Gene Pharma, Additional file 1: Table 2) were transfected into PBMCs by Lipofectamine RNAiMAX (Invitrogen, CAT# 13778-030) in accordance with the manufacturer’s instructions.

### Antibodies and reagents

Anti-RelB (CAT# 10544), anti-p52 (CAT# 37359), and rabbit anti-Flag (CAT# H2368) antibodies were purchased from Cell Signaling Technology. Mouse anti-Myc (CAT# 05-724), anti-p24 (CAT# MAB880-A), anti-RNA Pol II (CAT# 05-623B), normal mouse IgG (CAT# 12-371B) and normal rabbit IgG (CAT# 12-370) were from Millipore. Mouse anti-Flag (M2) (CAT# F1804), mouse anti-HA (CAT# H3663), and rabbit anti-EGFP (CAT# G1544) antibodies were obtained from Sigma. Anti-GAPDH (CAT# sc-32233), anti-Tubulin (CAT# sc-32293), rabbit anti-HA (CAT# sc-805), rabbit anti-Myc (CAT# sc-789), mouse anti-EGFP (CAT# sc-9996) and horseradish peroxidase-conjugated secondary antibodies (CAT# sc-2005 and sc-2004) were from Santa Cruz Biotechnology. FITC-conjugated goat anti-mouse IgG (CAT# 115-095-005) and TRITC-conjugated goat anti-rabbit IgG (CAT# 111-025-003) were from Jackson Immuno Research. RNase A (CAT# 20-297) was purchased from Millipore. DAPI (4′,6-diamidino-2-phenylindole) (CAT# D8417) and Phorbol 12-myristate 13-acetate (PMA) (CAT# P8139) were purchased from Sigma.

### Generation of stably transduced cell lines

First, HA-Tat or RelB was cloned into the pQCXIP. Then, retrovirus particles were prepared by transfecting HEK 293T cells (0.5 × 10^6^) with 1 μg pMLV-Gag-pol, 0.5 μg pVSV-G, and 1 μg pQCXIP DNA constructs. After 48 h, supernatants were collected and centrifuged at 3000 rpm to remove cell debris. HeLa, HEK 293T, Jurkat, or J-Lat A72 cells were infected with the harvested virus-like particles (VLPs) in the presence of 5 μg/ml polybrene by spinoculation at 450×*g* for 30 min at room temperature. Forty-eight hours after infection, cells were subcultured in selection medium containing 2 μg/ml puromycin (Sigma, CAT# P8833). Overexpressing efficiency was assessed by western blotting using specific antibodies. Design and construct sgRNA for RelB knockout (*RELB*^−*/*−^) are as follows: sgRNA1#: forward (5′-CACCGCCCGCGTGCATGCTTCGGTC-3′); reverse (5′-AAACGACCGAAGCATGCACGCGGGC-3′); sgRNA2#: forward (5′-CACCGTCGCCGCGTCGCCAGACCGC-3′); reverse (5′-AAACGCGGTCTGGCGACGCGGCGAC-3′). sgRNA was cloned into the pLentiCRISPR vector. To produce lentiviruses, pLentiCRISPR (with sgRNA cloned) was cotransfected into HEK 293T cells with the packaging plasmids pVSV-G and psPAX2. For each well of a 12-well plate, cells were infected with the harvested virus particles in the presence of 5 μg/ml polybrene by spinoculation at 450×*g* for 30 min at room temperature. Forty-eight hours later, cells were seeded into 96-well plates at the density of 0.5 cell/well with selection medium containing 2 μg/ml puromycin. After amplification for 2–3 weeks, the *RELB*^−*/*−^ cells were verified by western blotting.

### Viruses and infection

VSV-G pseudotyped HIV-1 (pNLENY1-ES-IRES and pNL4-3.Luc.env^−^) was produced by transfecting HEK 293T cells with proviral DNA constructs and pVSV-G plasmids. For VSV-G pseudotyped HIV-1, virus titer was determined by ELISA (Biomerieux) to quantify viral p24 amounts. For VSV-G pseudotyped HIV-1 infection, HeLa and Jurkat cells were spinoculated with VSVG-NL4-3.Luc.env^−^ (20 ng p24). Luciferase assays were performed 48 h’ post infection.

### Co-immunoprecipitation

For protein–protein interaction analysis, A total of 4 × 10^6^ cells were transfected with various plasmids using the PEI reagent. After 48 h, cells (1 × 10^7^–2.5 × 10^7^) were harvested and lysed in immunoprecipitation buffer (50 mM Tris [pH 7.4], 150 mM NaCl, 2 mM EDTA, 3% Glycerol, 1% Triton-X-100, protease inhibitor cocktail tablets complete, and EDTA-free [Roche]). Cell lysates were sonicated and centrifuged at 13,000×*g* for 10 min at 4 °C. Supernatants were incubated with antibodies (1 μg) for 3 h at 4 °C, then rotated with Protein A-agarose (Millipore, CAT# 16-125) for 3 h or overnight (for endogenous protein immunoprecipitation) at 4 °C. After being washed with lysis buffer for six times, the immunoprecipitated materials were boiled in 40 μl 2 × SDS loading buffer and subjected to western blotting (Ninety-five percent of cell extracts were used for the IP experiment, 1% of cell extracts were used as input for western blotting.).

### Western blotting

Cell lysates or immunoprecipitated materials were resolved by SDS-PAGE and transferred onto the PVDF membranes (GE Healthcare). The membranes were blocked with 5% nonfat milk, then incubated with primary antibodies (0.1–0.5 μg/ml) at 4 °C overnight. After incubation with either goat anti-rabbit or goat anti-mouse secondary antibody conjugated with horseradish peroxidase (HRP) (0.05–0.1 μg/ml), membranes were treated with enhanced chemiluminescence reagents (Millipore). Protein signals were detected by exposure to X-ray films.

### Immunofluorescence microscopy assay (IFA)

Indirect IFA was performed as previously described [30]. Cells grown on poly-lysine-coated glass slides were fixed with 4% (wt/vol) paraformaldehyde in PBS for 10 min, followed by permeabilized with 0.1% Triton X-100 in PBS for 10 min. After incubation in the blocking buffer containing 3% FBS and 6% skim milk, cells were stained with primary antibodies (5 μg/ml, 2 h at room temperature), followed by incubation with FITC- or TRITC-conjugated secondary antibodies (2 μg/ml, 45 min at room temperature). DAPI was utilized to stain nuclei. Images were captured using Leica TCS SP5 laser scanning confocal microscope [35].

### Luciferase assay

Cells were seeded on 12-well plates. The following day, they were transfected with reporter gene plasmid DNA along with the Renilla luciferase plasmid (phRluc-TK, Promega, CAT# E6921). Forty-eight hours after transfection, cells were harvested in lysis buffer, and luciferase assays were performed using the Dual-Luciferase reporter assay system (Promega, CAT# E1910) according to the manufacturer’s instructions. The relative luciferase activity was calculated by dividing the firefly luciferase activity by the Renilla luciferase activity. Three independent transfection experiments were performed.

### Chromatin immunoprecipitation (ChIP) assay and quantitative real-time PCR

The ChIP-qPCR assay was performed in TZM-bl cells. The DNA-IP assay was performed in HeLa transfected with pHIV-1-LTR-luc. ChIP-qPCR assay and DNA-IP assay were performed using the Upstate Biotechnology ChIP assay kit (Millipore, CAT# 17-371*RF*) according to the manufacturer’s protocol. In brief, formaldehyde was added to the culture medium at a final concentration of 1% and incubated at 37 °C for 10 min, and the chromatin was immunoprecipitated with antibodies or control IgG antibodies. One-tenth of the lysates was kept to measure the amount of DNA present in different samples before immunoprecipitation. DNA was purified from both the immunoprecipitated and preimmune samples, diluted from 1: 10 to 1: 10,000, and subjected to PCR and a real-time PCR assay to detect HIV-1 LTR DNA using the primer pair LTR forward (5′-TAGAGTGGAGGTTTGACAGCCG-3′) and LTR reverse (5′-GTACAGGCAAAAAGCAGCTG-3′). Glyceraldehyde-3-phosphate dehydrogenase (GAPDH) promoter DNA using the primer pair forward (5′-TACTAGCGGTTTTACGGGCG-3′) and LTR reverse (5′-TCGAACAGGAGGAGCAGAGAGCGA-3′) was also amplified from a ChIP assay that was performed with anti-RNA Pol II antibodies. The results served as a positive control. Real-time PCR was performed using FastStart Universal SYBR Green PCR Master Mix (Roche) according to the manufacturer’s instructions. Data were analyzed according to the ΔΔCt method and normalized to the input and IgG control.

### Flow cytometry

Cells were harvested and fixed in 1% formaldehyde. YFP/GFP positive cells were scored with a FACSCalibur flow cytometer and analyzed using the FlowJo software.

### Statistical analysis

Data were expressed as the mean ± standard deviation (SD) of three independent experiments, in which each assay was performed in triplicate. Data were compared using the unpaired two-tailed *t* test. *P *< 0.05 was considered significant, symbolized by *P < 0.05 and **P < 0.01. ns stands for not significant.

## Results

### HIV-1 Tat interacts with RelB

**Fig. 1:**
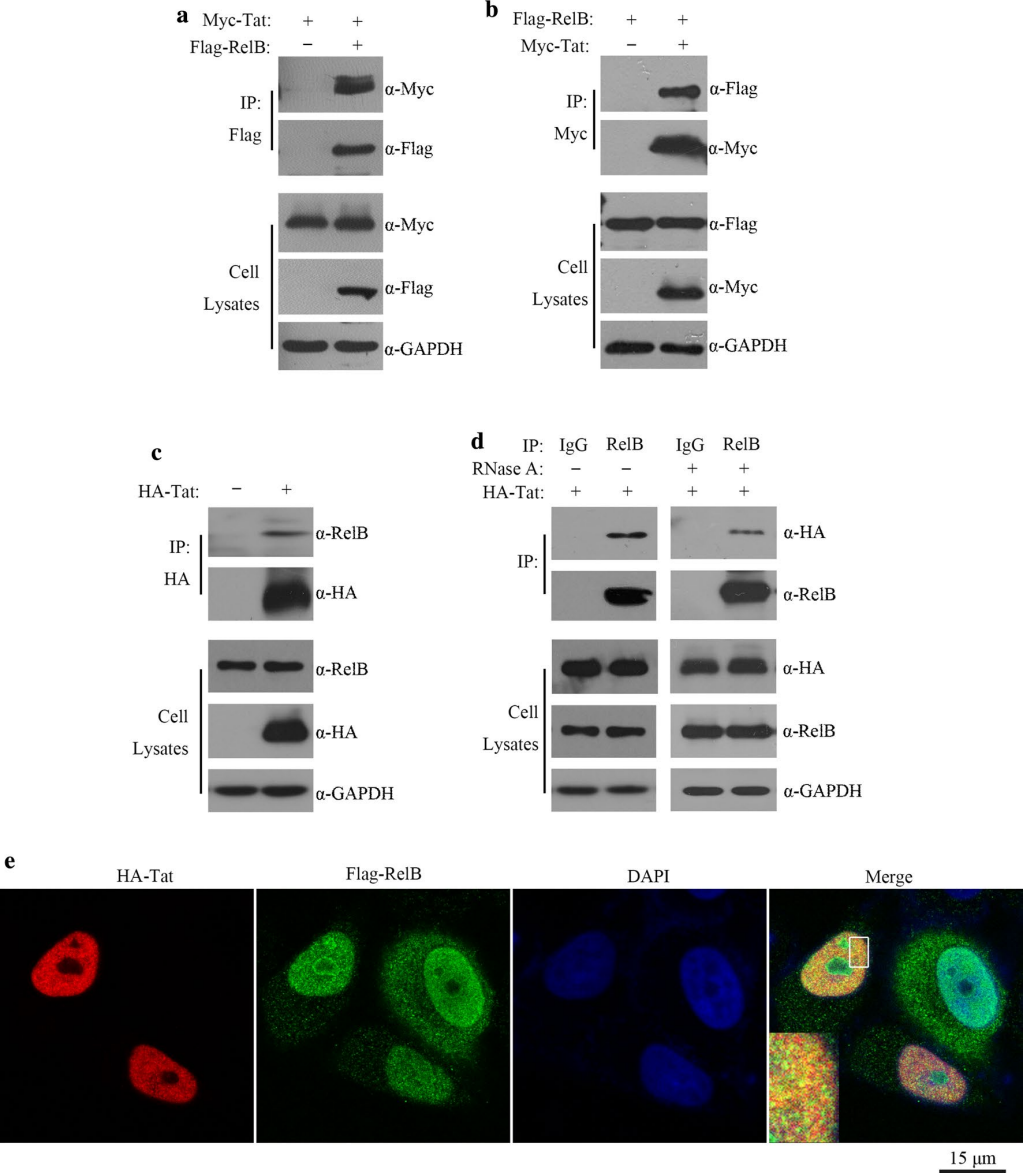
Interaction between HIV-1 Tat and RelB. **a**, **b** Myc-Tat (3 μg) was transfected into HEK 293T cells (4 × 10^6^) together with empty vectors (control) (3 μg) or pFlag-RelB (3 μg) Co-immunoprecipitation was performed with anti-Flag (**a**) or anti-Myc (**b**) antibodies. Samples of both cell lysates and immunoprecipitates were subjected to western blotting and probed with rabbit anti-Myc and anti-Flag antibodies. **c** Co-IP of endogenous RelB and ectopically expressed Tat. The lysate from HA-Tat-expressing HeLa cells (4 × 10^6^) was immunoprecipitated with mouse anti-HA antibodies, and the precipitated proteins were examined with western blotting. **d** Effect of RNases on the association of endogenous RelB and ectopically expressed Tat. Lysates (in the presence or absence of RNase A [5 μg/ml]) of HA-Tat expressing HeLa cells (4 × 10^6^) were immunoprecipitated with control rabbit IgG or rabbit anti-RelB antibodies. Samples from cell lysates and immunoprecipitates were subjected to western blotting. **e** Tat partially co-localizes with RelB. HeLa cells (0.1 × 10^6^) were transfected with HA-Tat (200 ng) and Flag-RelB (200 ng) plasmid DNA. Indirect IFA was performed to detect HA-Tat (with rabbit anti-HA antibody and TRITC-conjugated goat anti rabbit secondary antibody) and Flag-RelB (with mouse anti-Flag antibody and FITC-conjugated goat anti mouse secondary antibody). Nuclei were visualized with DAPI staining. Representative images are shown. The inset shows a higher magnification of the boxed area

To identify proteins that interact with HIV-1 Tat, we performed a yeast two-hybird screening with a human universal cDNA library as the prey and with HIV-1 Tat as the bait. Among 1 × 10^7^ cDNA clones screened, 21 clones showed interaction with Tat. One clone contained a 648 bp cDNA fragment of the RelB protein (236–452 aa) (GenBank accession no. NM_006509.2). We then performed experiments to validate this putative interaction between Tat and RelB. HEK 293T cells were co-transfected with Myc-Tat and Flag-RelB DNA, followed by co-immunoprecipitation with anti-Flag antibodies or anti-Myc antibodies. Flag-RelB was co-immunoprecipitated with Myc-Tat (Fig. 1a, b). We further tested whether Tat interacts with endogenous RelB. The results showed that Tat was precipitated with endogenous RelB in HeLa cells (Fig. 1c). As we know, Tat is an RNA-binding protein, it could indirectly interacted with other proteins by binding to RNA. In order to avoid the effect of RNA on protein interaction, we added RNase A into the buffer solution of IP experiments. As shown in Fig. 1d, endogenous RelB could still interact with Tat in the presence of RNase A. Further supporting the association between Tat and RelB, HA-Tat was found to partially co-localize with Flag-RelB in the nucleus (Fig. 1e). Together, these data demonstrate an association of Tat with RelB.

### Identification of protein sequences responsible for Tat and RelB interaction

**Fig. 2:**
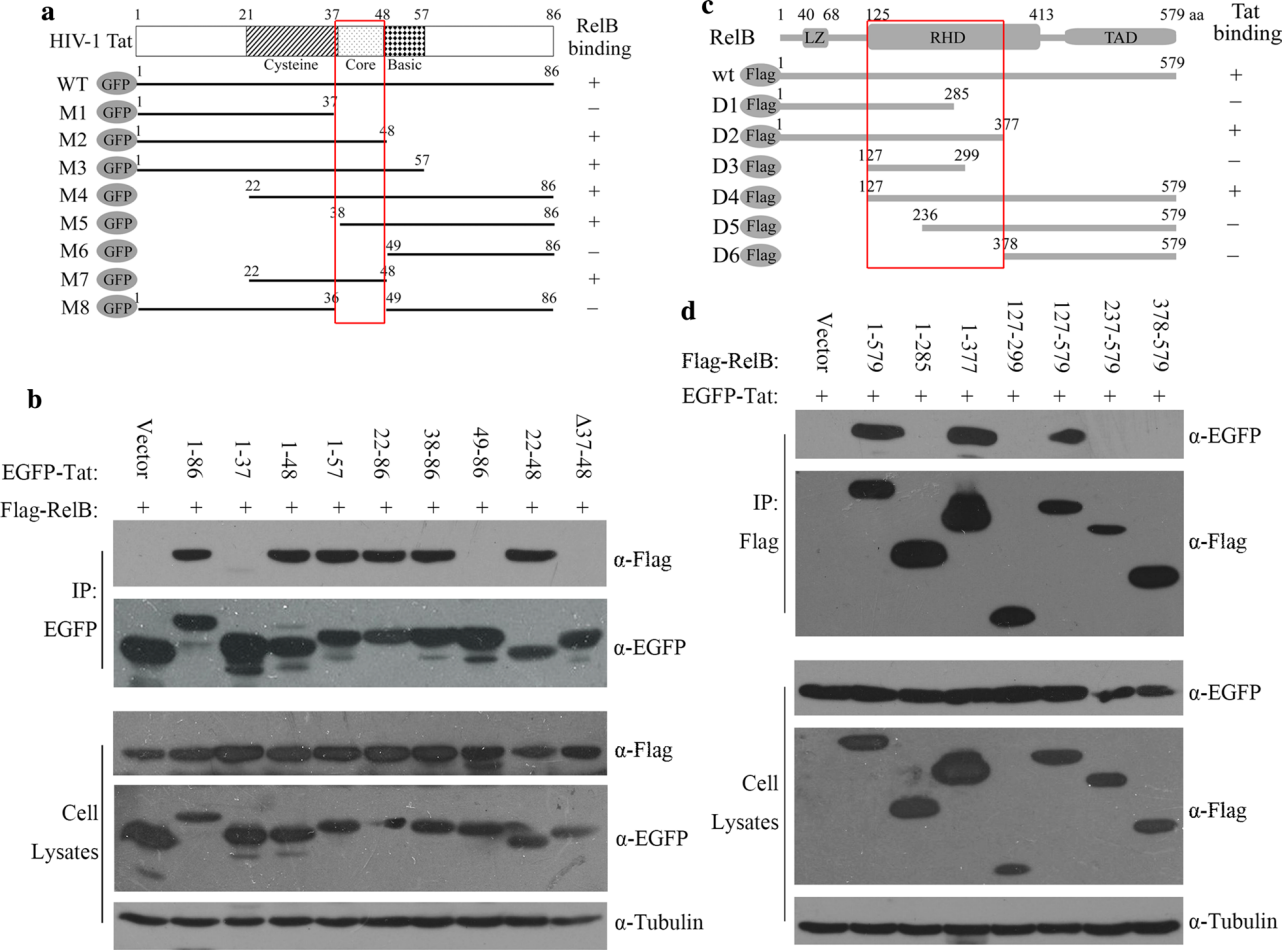
Identification of the domain responsible for Tat and RelB interaction. **a** Schematic representation of Tat and deletion mutants in the context of EGFP fusion proteins. The ability of each mutant to bind to RelB (see experimental results in Fig. 2b) is indicated on the right. The red box indicates the core domain that interacts with RelB. **b** pFlag-RelB (3 μg) was transiently transfected into HEK 293T cells (4 × 10^6^) together with the indicated wild type or truncated Tat DNA (3 μg). Tat proteins were immunoprecipitated (IP) (anti-EGFP) and immunoblotted with the indicated antibodies. **c** Schematic representation of RelB protein and its deletion mutants carrying a Flag tag. The ability of each mutant to bind to Tat (see experimental results in Fig. 2d) is shown on the right side. The red box partly covers the RHD domain of RelB, which binds Tat. **d** HEK 293T cells (4 × 10^6^) were transfected with pEGFP-Tat DNA (3 μg) and the indicated Flag-tagged deletion mutants of RelB (3 μg). RelB was immunoprecipitated (IP) (anti-Flag) and immunoblotted with the indicated antibodies

We next performed experiments to identify the RelB-binding domain in Tat. To this end, EGFP-Tat and a series of deletion mutants were constructed and co-transfected with Flag-RelB (Fig. 2a). The results showed that the core region of Tat (amino acid 37 to amino acid 48) is essential for binding to RelB (Fig. 2b, Additional file 2A). To map the Tat-binding domain in RelB, we constructed and co-transfected a series of Flag-RelB deletion mutants with EGFP-Tat, and found that the region between amino acid 127 and amino acid 377 in RelB was responsible for the interaction (Fig. 2d, Additional file 2B). Taken together, these results indicate that Tat interacts with RelB through a conserved core region of Tat and the Rel homology domain (RHD) of RelB. Notably, the RHD mediates RelB’s interaction with its partner proteins (e.g. p52), which guides the binding of RelB to the promoter of its target genes and their subsequent activation. This prompted us to examine whether Tat and RelB interaction is related to Tat-mediated transcription.

### RelB facilitates Tat-mediated HIV-1 LTR activation in TAR-independent manner

**Fig. 3:**
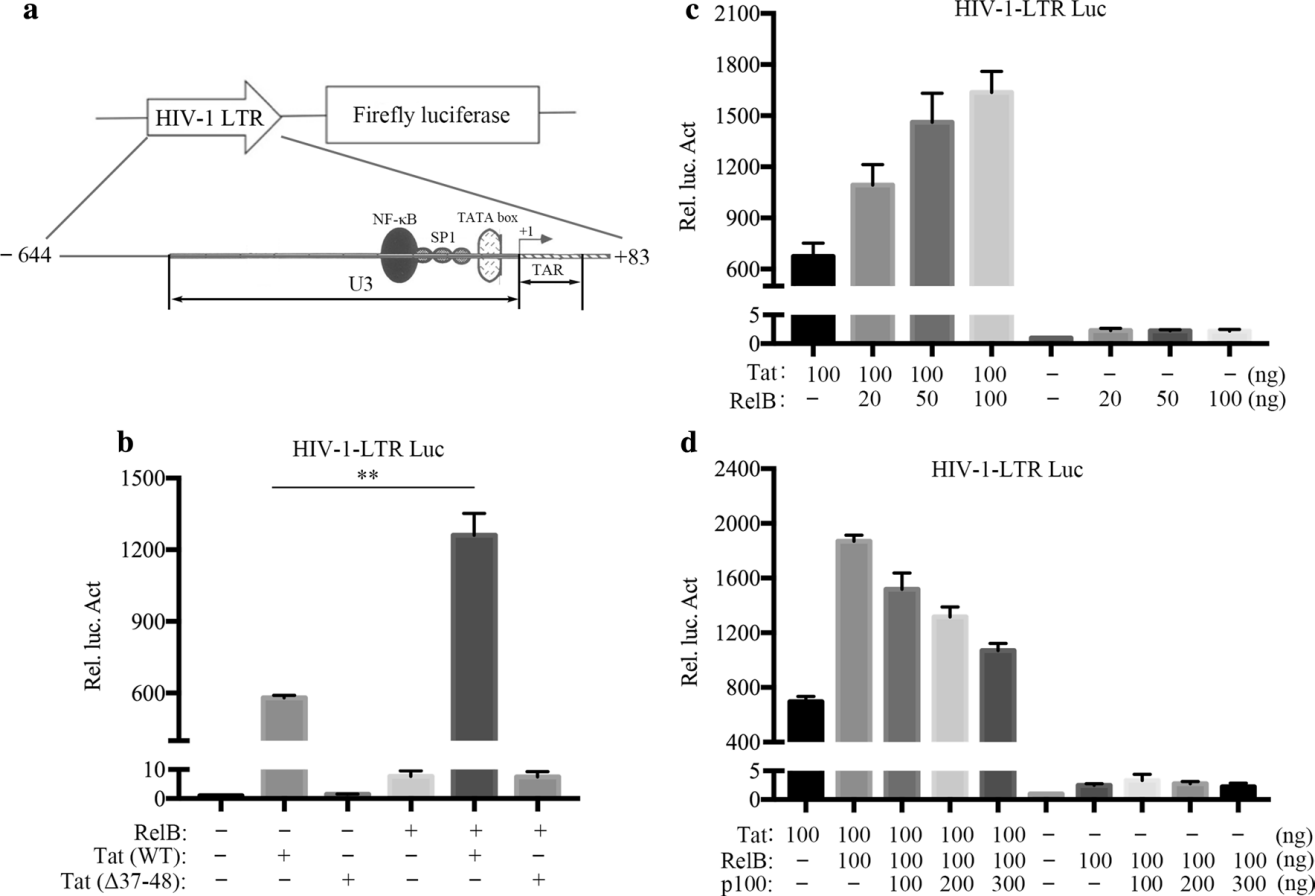
RelB enhances Tat transactivation of HIV-1 LTR. **a** Illustration of the luciferase reporter system. **b**–**d** HeLa cells (0.5 × 10^5^) were cotransfected with a reporter construct containing the HIV-1 LTR upstream of the luciferase gene (100 ng) and indicated plasmids (100 ng). To normalize transfection efficiency, phRluc-TK (1 ng) was co-transfected. Total DNA amounts were adjusted to 500 ng with an empty vector. After 48 h, luciferase activities were measured. The Rel. Luc. Act in the figure was calculated relative to normalized luciferase activity of pHIV-1-LTR-luc with Tat (with or without RelB/p100) and vectors co-transfected cells. The histogram shows mean ± SD of at least three independent experiments. *P < 0.05, **P < 0.01 (paired *t* test)

Since RelB is a transcriptional factor and can interact with Tat, we suspected that RelB may facilitate Tat-mediated activation of HIV-1 LTR. To test this hypothesis, we transfected HIV-1 LTR-driven firefly luciferase (pHIV-1-LTR-luc [30, 32]), contain the entire U3 and the first 83 nucleotides of R) (Fig. 3a) and RelB expression plasmids (pCMV-RelB) into HeLa cells in the presence or absence of the Tat expression plasmid (pCMV-Tat). The transactivation inactive Tat (Δ37–48 aa) was used as a negative control. Tat alone markedly increased the transcription of the LTR, expression of RelB further increased this activity by more than twofold. RelB itself had little effect on the HIV-1 LTR (Fig. 3b). In addition, RelB overexpression increased Tat-mediated transcriptional activity in a dose-dependent manner (Fig. 3c). Conversely, RelB inhibitor p100 prevented RelB from enhancing Tat-induced LTR transcription in a dose-dependent manner (Fig. 3d). These data suggest that RelB enhances Tat-induced LTR transcription.

**Fig. 4:**
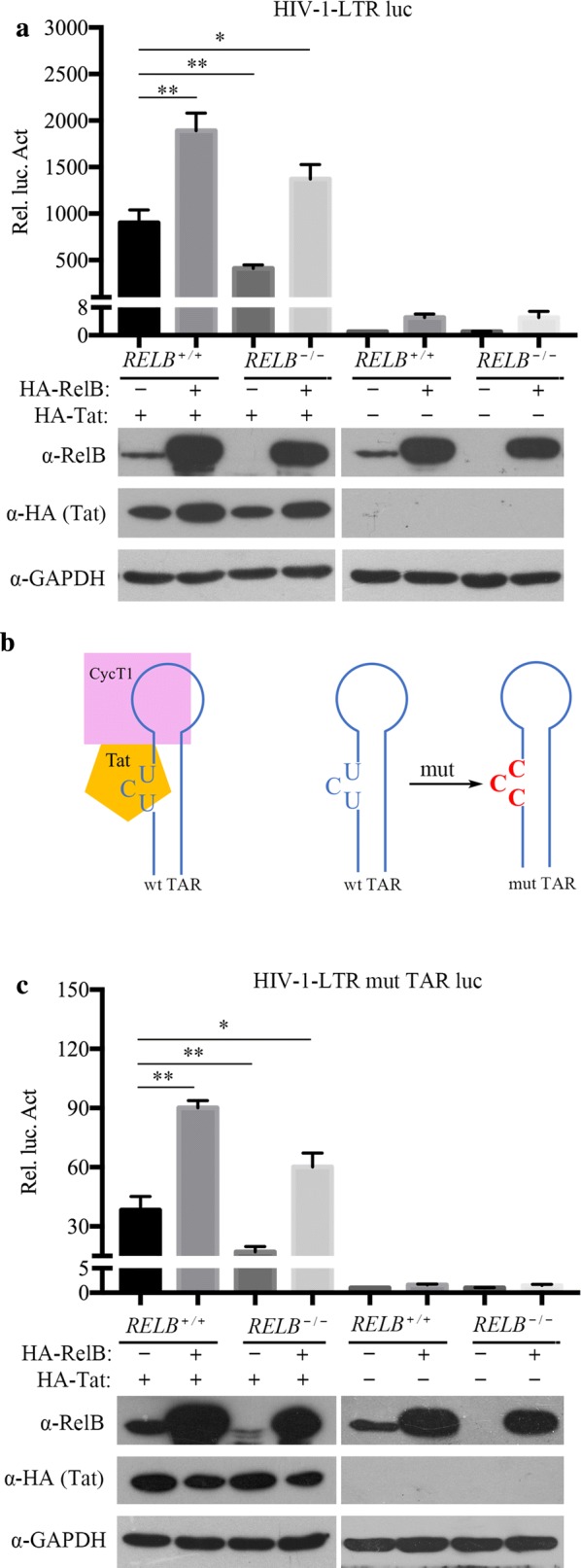
Endogenous RelB facilitates Tat-induced, TAR-independent HIV-1 LTR transactivation. **a** Control and *RELB*^−*/*−^ HeLa cells (0.1 × 10^6^) were transfected by indicated plasmids (pHA-Tat: 100 ng, pHA-RelB: 100 ng) and pHIV-1-LTR-luc luciferase reporter plasmids (100 ng). **b** Schematic representation of the CycT1-Tat-TAR ternary complex and TAR mutation. **c** Control and *RELB*^−*/*−^ HeLa cells (0.1 × 10^6^) were transfected with the indicated plasmids (pHA-Tat: 100 ng, pHA-RelB: 100 ng) and pHIV-1-LTR-luc luciferase reporter plasmids (100 ng). To normalize transfection efficiency, phRluc-TK (1 ng) was co-transfected. The Rel. Luc. Act in the figure was calculated relative to normalized luciferase activity of pHIV-1-LTR-luc with Tat (with or without RelB) and vectors co-transfected cells. The relative expression of RelB in the transfected cells was monitored by western blotting

Next, we examined the role of endogenous RelB in Tat-mediated transcriptional activation by knocking out RelB in HeLa cells using CRISPR-Cas9. The results showed that Tat-mediated activation of LTR promoter was significantly decreased (Fig. 4a). When RelB was ectopically expressed in RelB knockout (*RELB*^−*/*−^) HeLa cells, Tat activation was again increased (Fig. 4a).

Previous studies have reported that Tat can transactivate the LTR through a TAR-independent mechanism [8, 9, 12]. We next examined whether TAR is necessary for RelB to enhance Tat activation. It is known that Tat binds to the bulge of TAR [44]. We therefore mutated the bulge to abolish TAR-dependent Tat transactivation (Fig. 4b) [45]. Results in Fig. 4c show that RelB overexpression still increased Tat-mediated LTR-mutTAR activation, and that this activation was significantly decreased by RelB knockout.

### Tat and RelB are recruited to the HIV-1 promoter

**Fig. 5:**
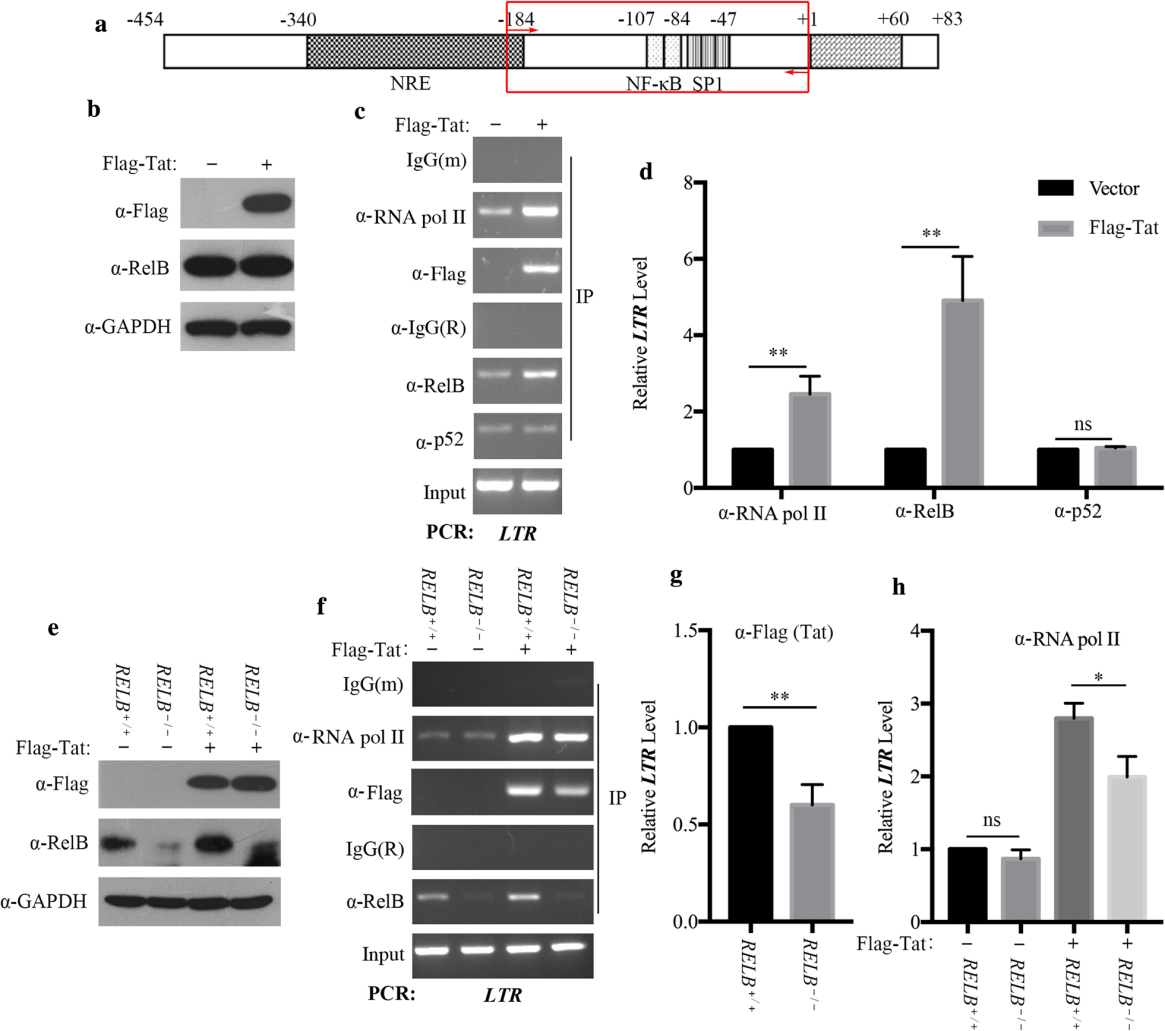
Association of RelB and Tat binding to the HIV-1 LTR. **a** Schematic illustration of the HIV-1 LTR and location of oligonucleotide primers. ChIP primers for HIV-1 LTR analysis were designed to span the enhancer region, including the duplicated κB enhancers (− 104 to − 81) and specificity protein 1 binding sites (SP1, − 79 to − 47). Primers for analysis of initiated HIV-1 transcripts were directed against TAR. **b**–**d** TZM-bl cells were transfected with pFlag-Tat (6 μg) or vector plasmids. The relative expression of Tat and RelB in the transfected cells was monitored by a western blot assay (**b**). Chromatin of the fixed TZM-bl cells (1 × 10^7^) was immunoprecipitated with the indicated antibodies. Samples were assessed for enrichment of HIV-1 LTR DNA by PCR (**c**). Relative levels of the LTR DNA that were co-precipitated with RNA Pol II, RelB, and p52 were quantified by real-time PCR performed in triplicate. The data are normalized to the input controls. Data are representative of three independent experiments (**d**). **e**–**h** Control and *RELB*^−*/*−^ TZM-bl cells were transfected with pFlag-Tat (6 μg) or vector plasmids (**e**). The co-precipitated LTR DNA was assessed by PCR (**f**). Tat (**g**) and RNA Pol II (**h**) associated HIV-1 LTR was quantified by real-time PCR

To confirm that Tat and RelB are recruited to the HIV-1 promoter, we performed chromatin immunoprecipitation assay and quantitative real-time PCR (ChIP-qPCR) using the TZM-bl cells. TZM-bl cells have an integrated HIV-1 LTR that drives the expression of the luciferase reporter gene. The primers used to amplify the − 204 to − 4 nt region covers the κB enhancers in the HIV-1 promoter (Fig. 5a). TZM-bl cells were transfected with or without Flag-Tat (Fig. 5b). As shown in Fig. 5c, d, immunoprecipitation with anti-Flag or anti-RelB antibodies enriched the LTR promoter sequence, suggesting the recruitment of Tat and RelB to the LTR. In addition, immunoprecipitation of Tat-stimulated samples with anti-RelB and anti-RNA Pol II antibodies led to marked increase in HIV-1 LTR DNA compared to the non-Tat control. This indicates the recruitment of both RelB and RNA Pol II to the LTR with Tat stimulation. Binding of p52 to the LTR did not change with Tat stimulation, suggesting that RelB may employ a unique mechanism to modulate the transactivation activity of Tat. We also examined the effect of Tat on the association of RNA Pol II with cellular promoter. ChIP experiment was thus performed to measure the association of RNA Pol II with the promoter of GAPDH gene. The results showed similar levels of GAPDH promoter DNA in both the control and Tat-transfected samples, suggesting that Tat does not affect RNA Pol II association with a constitutively transcribed cellular gene (Additional file 3A and B).

Tat and Rel family transactivators were reported to play a critical role in transcription complex assembly at the preinitiation step [46]. Given the recruitment of Tat and RNA Pol II to the HIV-1 LTR, we investigated the possibility that RelB might function as a transcriptional activator of the HIV-1 LTR, thereby reinforcing HIV-1 transactivation. To assess this possibility, we constructed the RelB knockout TZM-bl cell line and transfected it with Flag-Tat DNA (Fig. 5e). Results of ChIP assays with anti-RelB antibodies revealed enrichment of HIV-1 LTR DNA in control cells but not in RelB knockout cells (Fig. 5f). The recruitment of Tat to the HIV-1 LTR decreased as a result of RelB knockout (Fig. 5g), supporting the role of RelB in the recruitment of Tat to the HIV-1 LTR. With expression of Tat, knockout of RelB reduced the recruitment of RNA Pol II to the HIV-1 LTR. In the absence of Tat, knockout of RelB did not affect the recruitment of RNA Pol II (Fig. 5h). These results suggest that RelB assists the Tat-mediated recruitment of RNA Pol II to the LTR promoter.

### RelB facilitates Tat-mediated HIV-1 LTR activation depends on the NF-κB sites

**Fig. 6:**
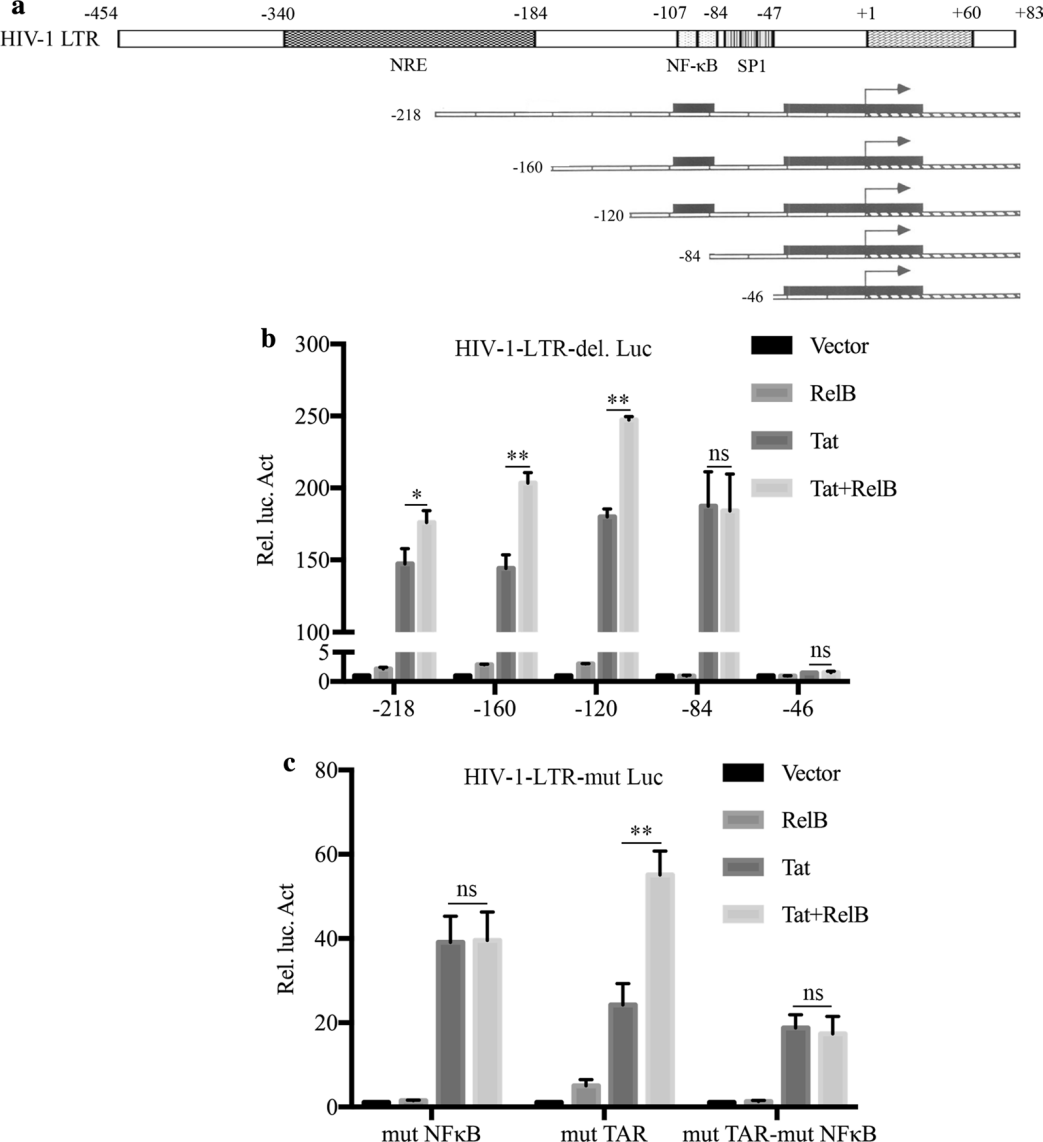
The κB elements are required for RelB to enhance Tat-mediated transcription of HIV-1 LTR. **a** Schematic representation of 5′-truncated pGL3-HIV-1-LTR-deletion reporter constructs. TAR (+ 1 to + 60), SP1 (− 79 to − 47), NF-κB (− 104 to − 81), and NRE (− 340 to − 184) sites are indicated. **b** Responsiveness of 5′-truncated pGL3-HIV-1-LTR reporter constructs to Tat and RelB. HEK 293T cells (0.2 × 10^6^) were cotransfected with each of the 5′-truncated pGL3-HIV-1-LTR-deletion reporter constructs (100 ng) in the presence of Tat-expressing plasmids, together with pCMV-RelB constructs or control vectors. **c** HeLa cells (0.1 × 10^6^) were transfected with the indicated HIV-1 LTR-Luc reporter plasmids (100 ng) and Tat-expressing plasmids (100 ng), together with pCMV-RelB constructs (100 ng) or control vectors. To normalize transfection efficiency, phRluc-TK (1 ng) was co-transfected. Luciferase assays were performed 48 h post transfection. The Rel. Luc. Act in the figure was calculated relative to normalized luciferase activity of pHIV-1-LTR-luc with Tat (with or without RelB) and vectors co-transfected cells

To identify the key sites of RelB enhances Tat-mediated HIV-1 LTR activation, we generated a series of deletions of the HIV-1 LTR (Fig. 6a). These LTR-mutated reporter plasmids were individually transfected into HEK 293T cells with pCMV-Tat and pCMV-RelB. The results showed that deletion of NF-κB-binding sequence (− 107 to − 84) eliminated the enhancing effect of RelB on HIV-1 activation in the absence and in the presence of Tat (Fig. 6b). To verify the roles of the NF-κB-binding sequences in the transcription of the LTR, we generated mutNFκB (Additional file 4), mutTAR, and mutTAR-mutNFκB reporter plasmids, and tranfected each of them into HeLa cells with Tat and RelB. As shown in Fig. 4c, the mutTAR promoter activity increased when Tat was co-expressed with RelB (Fig. 6c). In contrast, co-expressing RelB with Tat had little effect on expression of luciferase reporter from the mutNFκB and mutTAR-mutNFκB viral promoters (Fig. 6c). The results suggest that RelB increases Tat-mediated activation of the HIV-1 LTR in a NF-κB element-dependent and TAR-independent manner.

### NF-κB elements are essential for RelB to enhance Tat recruitment to the LTR

**Fig. 7:**
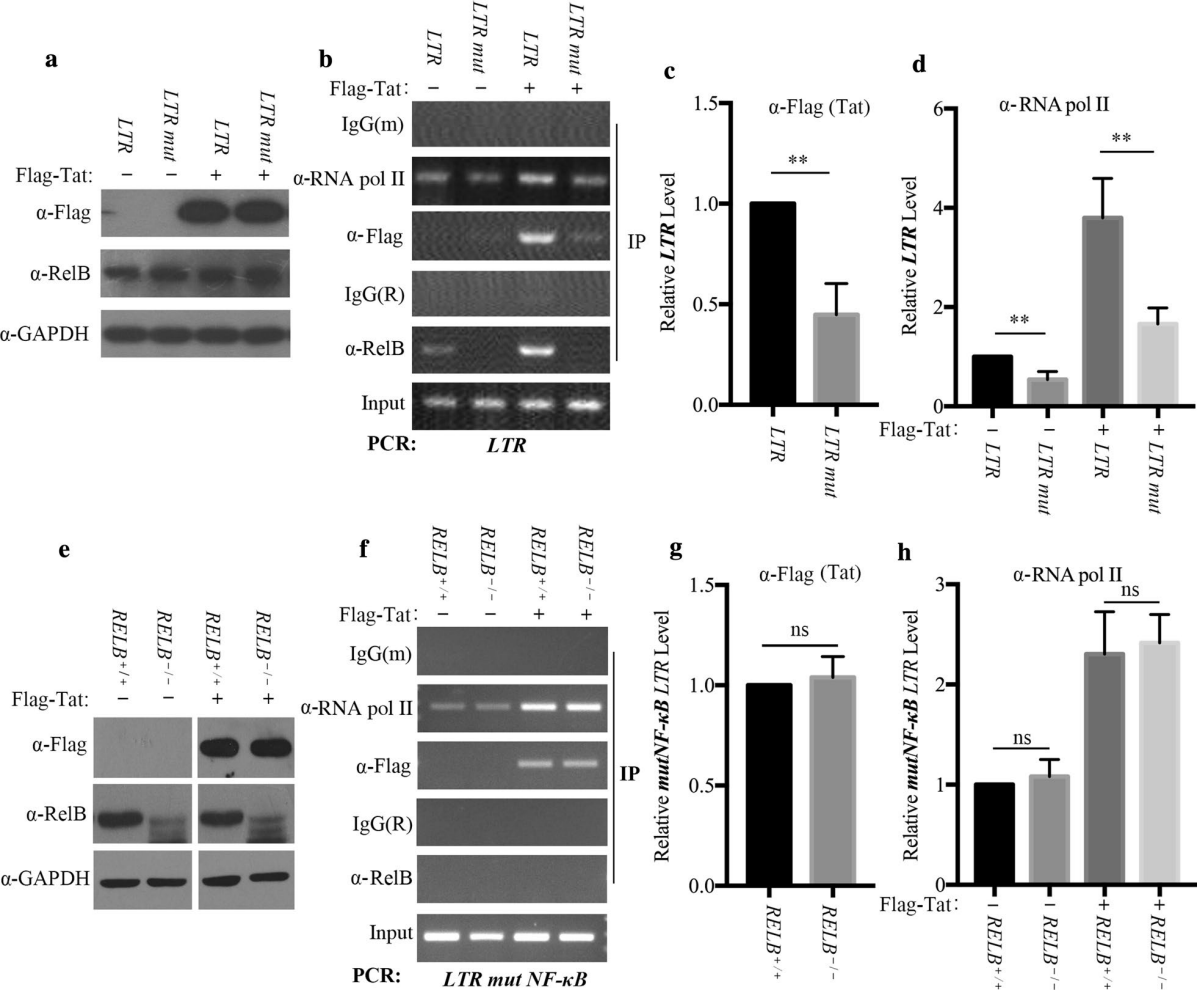
NF-κB elements are essential for RelB to enhance Tat recruitment to the LTR. **a–d** HeLa cells (1 × 10^7^) were transfected with pHIV-1-LTR-luc (6 μg) or pHIV-1-LTR mutNF-κB (6 μg), together with pFlag-Tat (6 μg) or vector plasmids. The relative expression of Tat and RelB in the transfected cells was monitored by western blots (**a**). Chromatin of the fixed cells was immunoprecipitated with the indicated antibodies. Samples were assessed for enrichment of HIV-1 LTR DNA by PCR (**b**). Tat (**c**) and RNA Pol II (**d**) associated HIV-1 LTR DNA was quantified by real-time PCR. (E to H) Control and *RELB*^−*/*−^ HeLa cells (1 × 10^7^) were transfected with pHIV-1-LTR mutNF-κB (6 μg), together with pFlag-Tat (6 μg) or vector plasmids (**e**). Samples were assessed for enrichment of HIV-1 LTR DNA by PCR. **f** Tat (**g**) and RNA Pol II (**h**) enriched HIV-1 LTR DNA was quantified by real-time PCR

To determine the effect of NF-κB elements on the recruitment of RelB and Tat onto the HIV-1 promoter, we transfected HIV-1 LTR or HIV-1 LTR-mutNFκB reporter plasmids into HeLa cells, together with Flag-Tat (Fig. 7a). Results of DNA-IP assays showed that mutation of the NF-κB elements significantly diminished RelB recruitment (Fig. 7b), as well as the recruitment Tat to the HIV-1 promoter (Fig. 7c). As a result, recruitment of RNA Pol II to the LTR was impaired (Fig. 7d). To further examine the role of RelB in recruiting Tat to the mutNF-κB HIV-1 promoter, we transfected HIV-1 LTR-mutNFκB DNA together with Flag-Tat plasmids into RelB knockout HeLa cells (Fig. 7e). As shown in Fig. 7f and G, RelB knockout did not affect Tat enrichment onto LTR-mutNFκB, nor the recruitment of RNA Pol II (Fig. 7f, h). Taken together, we conclude that the NF-κB elements are essential for RelB to enhance Tat recruitment to the LTR.

### RelB enhances HIV-1 gene expression

**Fig. 8:**
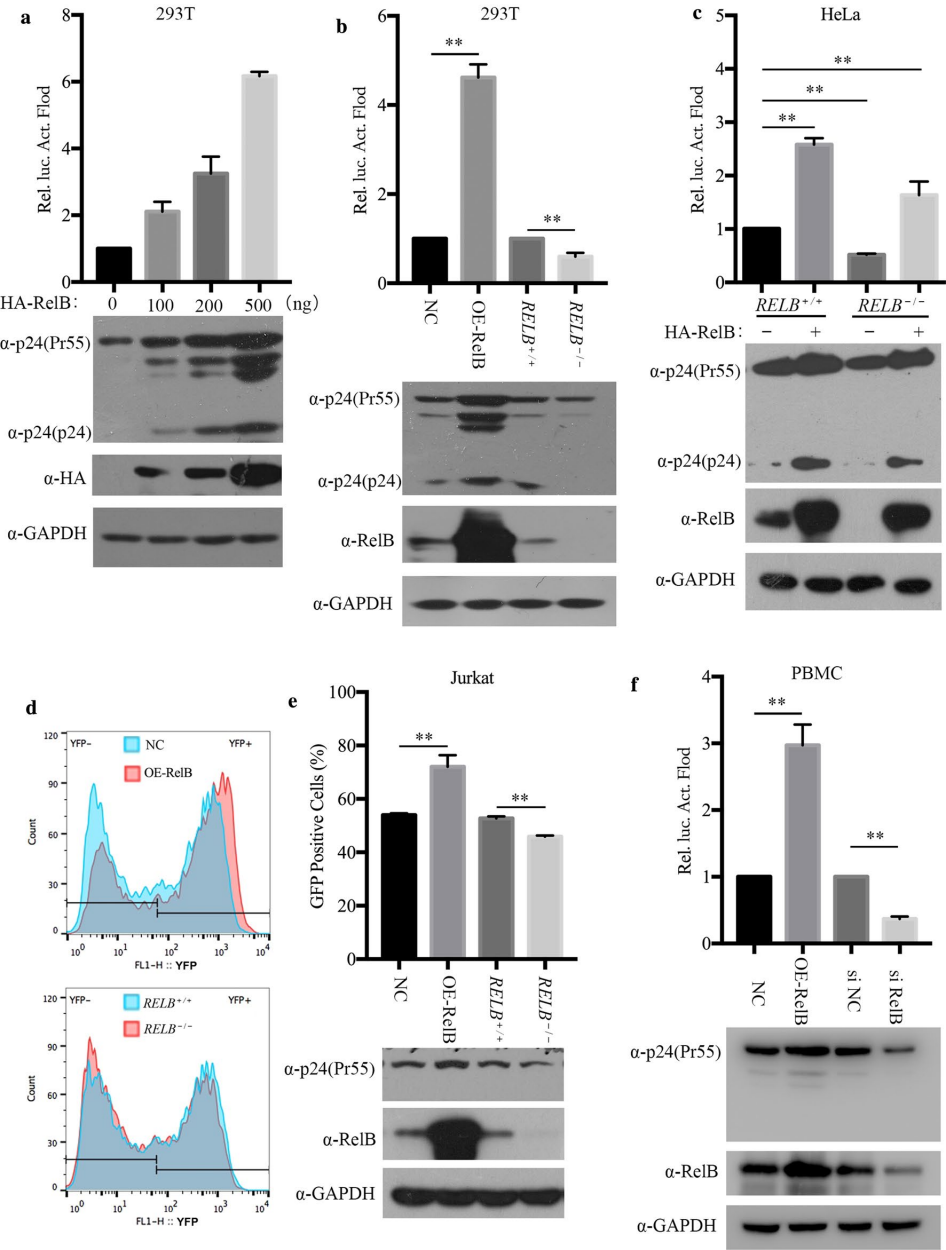
RelB increases HIV-1 gene transcription. **a** HEK 293T cells (0.5 × 10^6^) in a 6-well plate were transfected with increasing amounts of pHA-RelB, together with pVSV-G (0.3 μg) and pNL4-3.Luc.env^−^. (1 μg). Forty-eight hours post transfection, cells were collected for western blotting and luciferase activity analysis. **b** Stable RelB-overexpressing cells, *RELB*^−*/*−^ cells, and control HEK 293T cell lines (0.5 × 10^6^) were transfected with pVSV-G (0.3 μg) and pNL4-3.Luc.env^−^ (1 μg). Western blotting and Luciferase activity essays were performed to assess viral gene expression. **c** Control and *RELB*^−*/*−^ HeLa cells were transfected with pHA-RelB (500 ng) or vector DNA. Twenty-four hours post transfection, cells were infected with VSVG-NL4-3.Luc.env^−^ equivalent to 20 ng p24 for 40 h. Cells were collected for luciferase assay and western blotting analyses. **d** and **e** RelB overexpressing or *RELB*^−*/*−^ Jurkat cells were infected with VSVG-NLENY1-ES-IRES equivalent to 20 ng p24 for 48 h. Gating on live Jurkat cells was based on size (FSC-H) and granularity (SSC-H). YFP positive cells were scored by flow cytometry 48 h after infection (**d**). Results from three independent experiments were summarized, and cells were collected for western blotting analysis (**e**). **f** PBMCs (2 × 10^6^) were infected with RelB-expressing VLPs, or transfected with siRNA. After 24 h, infection with VSV-G pseudotyped NL4-3.Luc.env^−^ (equivalent to 500 ng p24) was performed by spinoculation at 300×*g* for 30 min. After 24 h, whole cell lysates were examined in luciferase assay and western blotting with the indicated antibodies

HIV-1 Tat is essential for HIV-1 production and replication. We have shown that RelB facilitates Tat-mediated activation of the HIV-1 LTR. We therefore hypothesized that RelB-mediated Tat activation increases HIV-1 gene transcription. To test this hypothesis, we used pseudotyped HIV-1 to examine the effect of RelB on HIV-1 gene transcription. We transfected HIV-1 DNA clone pNL4-3.Luc.env^−^ and pVSV-G together with increasing amounts of HA-RelB into HEK 293T cells. As shown in Fig. 8a, the levels of p24 and the enzyme activity of luciferase from these cells increased with RelB expression in a dose-dependent manner. To confirm this observation, we generated a stable RelB overexpresion HEK 293T cell line and a RelB knockout HEK 293T cell line. These cell lines were transfected with HIV-1 DNA clone pNL4-3.Luc.env^−^ and pVSV-G. Results showed that overexpression of RelB increased the expression of HIV-1 gene, while in the absence of RelB, viral expression diminished (Fig. 8b). These results indicate that RelB is required for HIV-1 transcription.

We next asked whether endogenous RelB has any effect on the transcription of HIV-1 during viral infection. To answer this question, we used VSV-G pseudotyped NL4-3.Luc.env^−^ to infect HeLa cell lines and measured luciferase activity 40 h post infection. The results showed that knockout of RelB decreased HIV-1 p24 levels and reduced HIV-1 gene expression. Enzyme activity of luciferase of infected cells was significantly increased with the restoration of RelB expression in RelB knockout HeLa cells, which corroborated the effect of RelB overexpression on HIV-1 gene expression (Fig. 8c) (Additional file 4).

We further confirmed these findings in other HIV-1 permissive cell line by generating Jurkat cell lines that either overexpressed RelB or had RelB knocked out. We infected these Jurkat cells with VSV-G pseudotyped NLENY1-ES-IRES. pNLENY1-ES-IRES is a proviral DNA clone and express yellow fluorescent protein (YFP) from HIV-1 LTR promoter [32, 37]. The YFP sequence was inserted between the env and nef ORFs. An internal ribosome entry sequence (IRES) was inserted upstream of nef to direct the expression of Nef (Additional file 5) [35]. We scored YFP positive cells by flow cytometry. Similar to the effect in HeLa cells, RelB overexpression increased the number of HIV positive cells, and RelB knockout reduced the number of HIV-1 positive cells (Fig. 8d, e). To validate this observation, we isolated PBMCs from healthy donors and examined effect of RelB on HIV-1 gene transcription. Again, RelB overexpression enhanced HIV-1 gene expression, and RelB knockdown reduced HIV-1 gene expression (Fig. 8f).

### RelB facilitates reactivation of HIV-1 provirus in latently infected cells

**Fig. 9:**
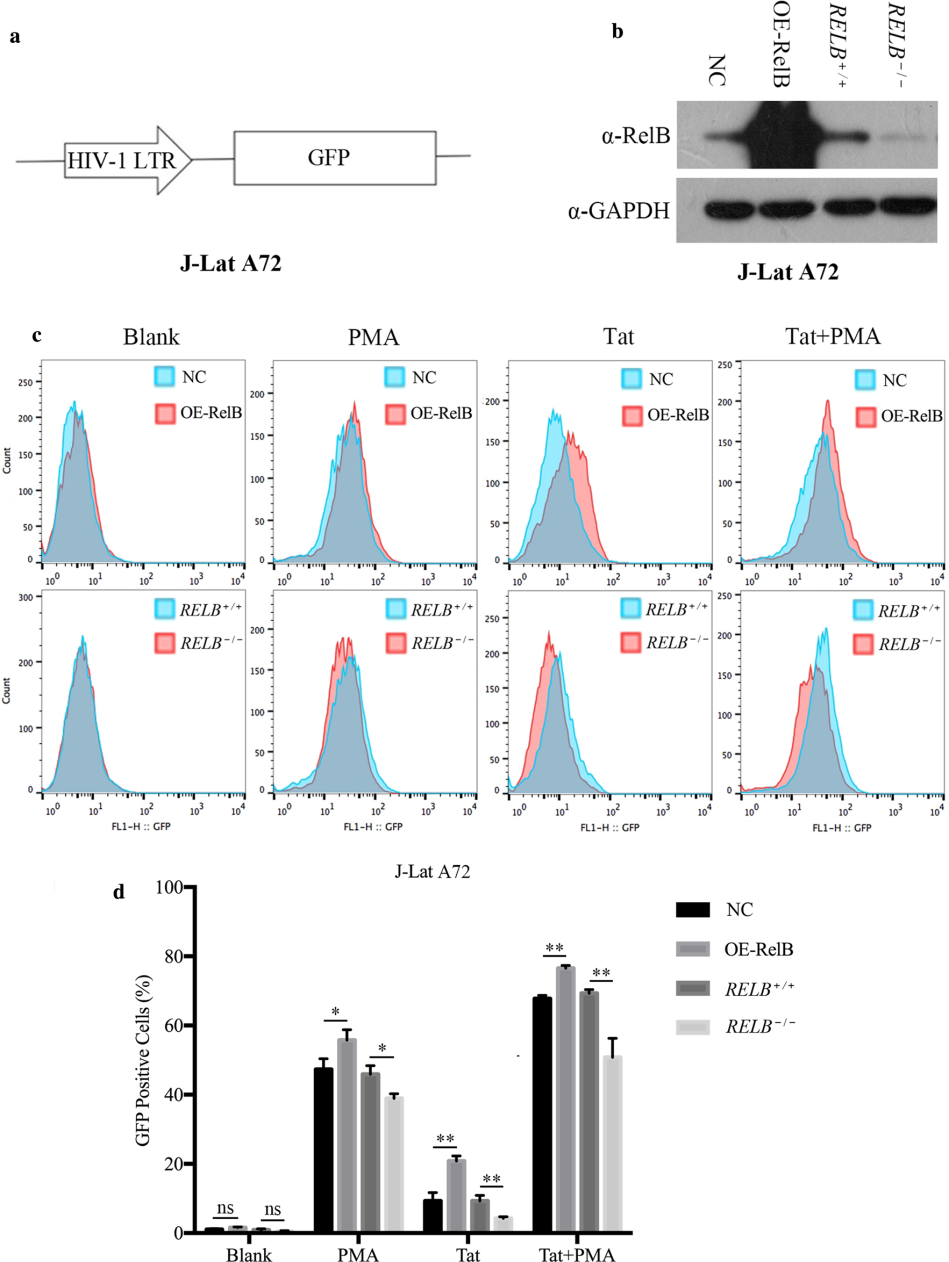
Requirement of RelB for the activation of latent HIV-1 in J-Lat A72 cells. **a** Illustration of the J-Lat A72 latency model. **b** Western blots to examine the overexpressed RelB or RelB knockout in J-Lat A72 cell lines. **c** RelB enhanced Tat-mediated activation of latent HIV-1 in J-Lat A72 cells. J-Lat A72 cells (2 × 10^6^) were transduced with VSV-G pseudotyped lentiviral VLPs expressing Tat. Twenty-four hours post infection, cells were treated with 10 nM PMA or DMSO for 24 h, then subjected to flow cytometry to detect GFP expressing cells using a FACSCalibur flow cytometer. Data were analyzed using the FlowJo software. **d** Analysis of HIV-1 LTR transcriptional activation by flow cytometry. Results from three independent experiments were summarized

Given that RelB enhances HIV-1 transcription, we postulated that RelB may play a role in reactivating HIV-1 latency through its interaction with Tat. J-Lat A72 harbors latently integrated HIV-1 LTR-GFP proviruses (Fig. 9a) [42, 43]. To test the effect of RelB on HIV-1 proviruses, we generated RelB overexpression and knockout (using CRISPR-Cas9) J-Lat A72 cell lines (Fig. 9b). We used Tat-containing virus-like particles (VLPs) or PMA (positive control) to stimulate these cells. GFP positive cells were scored by flow cytometry (Fig. 9c). RelB overexpression increased J-Lat A72 reactivation in addition to Tat enhancement, while RelB knockout decreased J-Lat A72 reactivation in the presence of Tat. Similar observations were observed in PMA stimulation but the effect was less pronounced. When both Tat and PMA were used to stimulate these cell lines, the role of RelB in activating HIV-1 latency was more prominent (Fig. 9d). These data suggest that endogenous RelB plays a role in HIV-1 proviruses in latently infected cells.

## Discussion

**Fig. 10:**
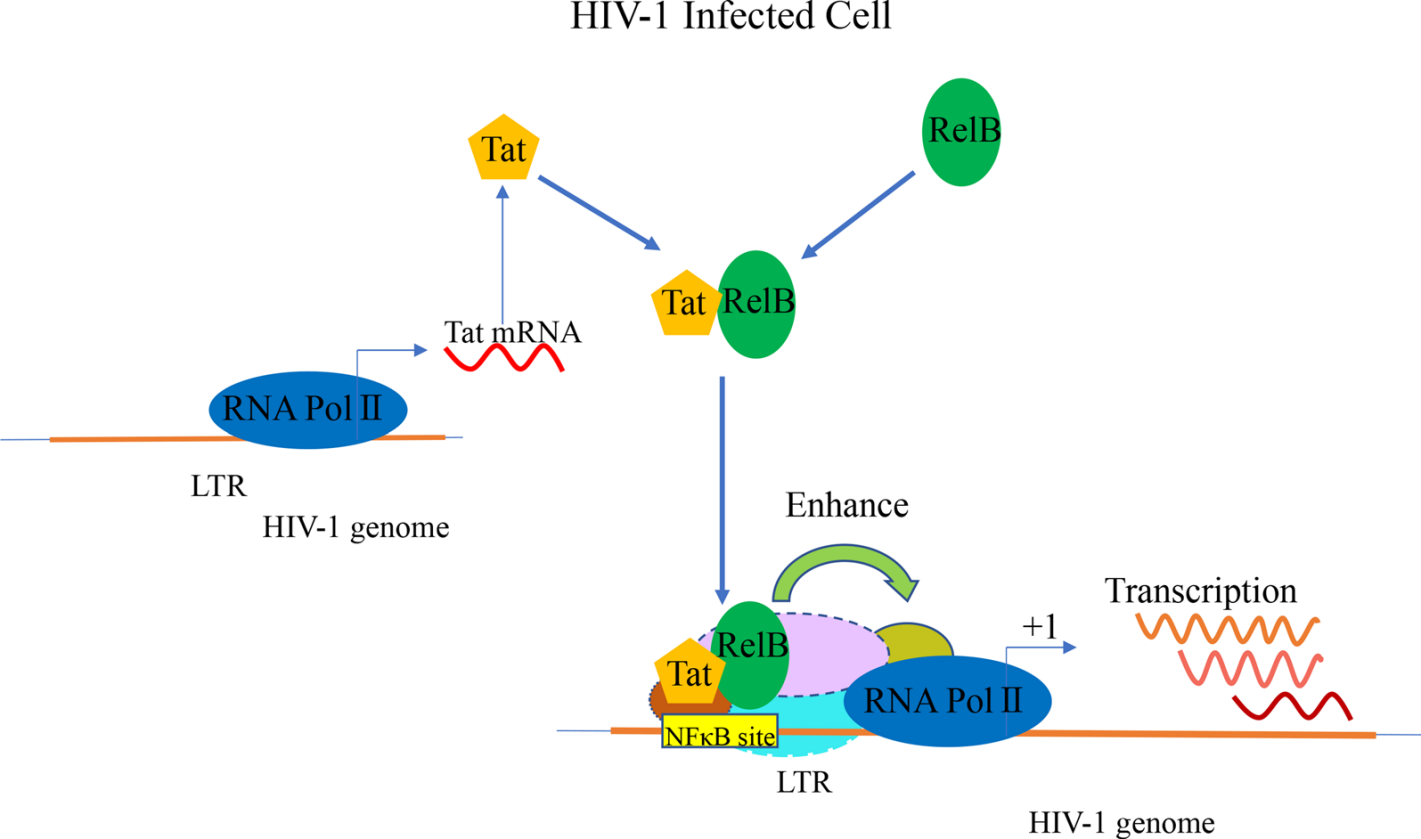
Model of HIV-1 transcriptional activation by Tat and RelB. HIV-1 Tat interacts with RelB. RelB and Tat are recruited to LTR DNA to assist transcription, which increases recruitment of RNA Pol II to the LTR and leads to greater expression of viral genes

Several lines of evidence suggest that TAR-independent Tat function requires specific sequences of the HIV-1 enhancer and promoter, potentially through interactions with cellular DNA binding proteins [46]. Structural studies indicate that Tat is a small protein (86–101 aa). By itself, Tat lacks a defined three-dimensional folding with a stable conformation, making it an intrinsically disordered protein [47]. However, Tat is able to form tight binding with it co-factors, such as the TAR element and Cyclin T1, as a result of its structural plasticity that allows Tat to adopt different conformations upon interaction with different partners [48–51]. In this study, we report that Tat interacts with RelB, and they are recruited to the LTR, which leads to increased recruitment of RNA Pol II to the LTR and greater transcription of viral genes (Fig. 10). Additionally, the RHD of RelB and the core region of Tat are found to be key to their interaction. These findings agree with previous reports showing that Tat contains structural motifs homologous to the N-terminal domain of the mouse and human NF-κB p50 subunit, which belongs to the p53 superfamily of transcription factors [22].

The formation of a NF-κB p50/RelB/DNA complex suggests that RelB may recognize more diverse κB sequences [52]. In this study, RelB efficiently increased Tat-dependent transactivation of the HIV-1 LTR. These data indicate that RelB is a potential Tat coactivator that facilitates the interaction between Tat and the transcription machinery on the HIV-1 LTR without TAR. Previous studies have suggested that Tat may be involved in facilitating and regulating RelA transcriptional activity [23, 53], but did not investigate whether Tat binds with RelB. One study reported that Tat induced RelB expression which inhibits cytokine production, possibly through the formation of transcriptionally inactive RelB/RelA complexes [54]. In our research, we found that RelB enhances Tat recruitment to the LTR via NF-κB elements and Tat increased the recruitment of RelB and RNA Pol II to the LTR. With RelB knockout, the recruitment of Tat to the LTR reduced, RNA Pol II complex assembling to the LTR also diminished. These results support an important role for RelB in the regulation of LTR transcription initiation. Moreover, we used pseudotyped HIV-1 to examine the effect of RelB on HIV-1 gene transcription. The result showed that RelB enhanced HIV-1 gene expression during HIV-1 infection. Based on these data, we speculate that in HIV-1 infected cells, Tat and cellular RelB form a transcriptional complex which recognizes and binds to the NF-κB elements of the LTR and facilitates the recruitment of other coactivators to ultimately initiate LTR transcription.

Tat establishes interactions with many host proteins to promote HIV-1 propagation and HIV-1 pathogenesis. Tat also affects both the establishment and reversal of latency [55]. For example, the deficiency of Tat protein contributes to latency mechanisms together with the deficiency of transcriptional factor NF-κB or NFAT, condensed chromatin structure, and epigenetic regulation [56–59]. It is possible that Tat facilitates the interaction of RelB and LTRs through chromatin structural remodeling in the LTR region. Our study support that, once Tat is produced, both transcription initiation and elongation are enhanced, which sustains continuous proviral gene expression. The interaction between RelB and Tat facilitates the assembly of transcription complexes on the LTR promoter (Fig. 10). We used the J-Lat A72 HIV-1 latency model to investigate the potential role of κB-specific transcriptional activators in the maintenance of viral transcription. Overexpressing Tat reactivated J-lat A72 cells, and RelB facilitated this reactivation. Based on our findings, RelB and Tat interaction contributes to proviral latency reactivation.

Similar to Tat, RelA stimulates transcription by recruiting P-TEFb to the initiating RNA Pol II [18, 60, 61]. Future work is needed to identify the specific mechanisms by which RelB binding regulates HIV-1 infection and the reactivation of latent proviruses.

Our study has discovered one more cellular factor that participates in the regulation of HIV-1 gene expression, which adds one more layer to the complex molecular mechanisms that center around Tat. It is unknown how these mechanisms and diverse molecular interactions function together. One possible scenario is that one mechanism may be more dominant over the other in a specific cell type, such as different subtypes of T cells, macrophage, dendritic cells, astrocytes and others, which depends on the availability and abundance of specific factors. It is also possible that HIV-1 needs several mechanisms to operate in concert to acquire an optimal level of viral gene expression, in response to different external stimuli. More studies are warranted to obtain further insights in this regards.

## Conclusions

In summary, our results demonstrate that Tat interacts with RelB, RelB in turn enhances TAR-independent transactivation of HIV-1 gene expression. RelB plays an important role in Tat-mediated κB-site-dependent HIV-1 initial transcription. Thus, we have identified a TAR-independent, NF-κB-dependent step in the recruitment of RNA Pol II to the HIV-1 promoter and demonstrated that RelB is a critical factor in the regulation of HIV-1 transcription (Fig. 10). Our findings may inspire the development of new therapies that either prevent the reactivation of latent proviruses or stimulate reactivation and subsequently clear latently infected cells.

## Additional files

**Additional file 1.** Table 1 List of primers used in mutagenesis and cloning. Table 2 List of siRNAs used in RNAi.

**Additional file 2.** Identification of Tat and RelB interaction minimal domains. (A) pFlag-RelB (3 μg) was transiently transfected into HEK 293T cells (4 × 10^6^) together with the wild type Tat or truncation mutants (3 μg). Tat proteins were immunoprecipitated (IP) (anti-EGFP), and immunoblotted with the indicated antibodies. (B) HEK 293T cells (4 × 10^6^) were transfected with pEGFP-Tat (3 μg) and the indicated Flag-tagged deletion mutants of RelB (3 μg). RelB was immunoprecipitated (IP) (anti-Flag), and immunoblotted with the indicated antibodies.

**Additional file 3.** Immunoprecipitation of *GAPDH*-associated RNA Pol II was not Tat dependent. (A) TZM-bl cells were transfected with pFlag-Tat (6 μg) or vector plasmids. Fixed chromatin in these cells was immunoprecipitated with the anti-RNA Pol II antibodies. Samples were assessed for enrichment in transcriptionally active control DNA (GAPDH) by UV visualization of PCR products in an ethidium bromide-stained agarose gel. (B) Relative levels were quantified by real-time PCR analysis performed in triplicate and normalized to input controls.

**Additional file 4.** Model of mutNFκB reporter plasmids. NF-κB mutation of the HIV LTR reporter plasmids were generated using PCR-based mutagenesis.

**Additional file 5.** Schematic presentation of the NLENY1-ES-IRES proviral DNA.
