# Supplementary material for: Cellular RelB interacts with the transactivator Tat and enhance HIV-1 expression
Source: Retrovirology. 2018 Sep 21;15:65. doi: 10.1186/s12977-018-0447-9 (PMC6150996; doi:10.1186/s12977-018-0447-9)
Supplement: Supplementary file 1 — Additional file 1. Table 1 List of primers used in mutagenesis and cloning. Table 2 List of siRNAs used in RNAi. [file 12977_2018_447_MOESM1_ESM.docx]

**Additional files 1.**

Table 1 List of primers used in mutagenesis and cloning.

| Name | Sequence (5’-3’) |
| --- | --- |
| QC-RelB-F | TTTGCGGCCGCATGCTTCGGTCTGGG |
| QC-RelB-R | CATGAATTCCTACGTGGCTTCAG |
| QC-Tat-F | ACACTCGAGATGGAGCCAGTAG |
| QC-Tat-HA-R | CCGGAATTCTTAAGCGTAATCTGGAACATCGTATGGGTAATCGACCGGATCTGTC |
| Myc-Tat-F | GGACCATGGAAATGGAGCCAGTAGATCC |
| Myc-Tat-R | AACGAATTCCTAATCGACCGGATCTGTCTCTG |
| EGFP-Tat-F | CCGCTCGAGCTATGGAGCCAGTAGATCCTAG |
| EGFP-Tat22-F | CCGCTCGAGCTTGTACCAATTGCTATTG |
| EGFP-Tat37-F | CCGCTCGAGCTTGTTTCATAACAAAAGC |
| EGFP-Tat49-F | CCGCTCGAGCTAGGAAGAAGCGGAGACA |
| TatΔ (37-48) F | TCATTGCCAAGTTAGGAAGAAGCGGA |
| TatΔ (37-48) R | TCCGCTTCTTCCTAACTTGGCAATGA |
| EGFP-Tat37-R | AACGAATTCTTAACAAACTTGGCAATG |
| EGFP-Tat48-R | AACGAATTCTTAGCCATAGGAGATGCCTAAG |
| EGFP-Tat-R | AACGAATTCCTATTCCTTCGGGCCTGT |
| EGFP-Tat (37-48)-F | TCGAGCTTGTTTCATAACAAAAGCCTTAGGCATCTCCTATGGCTAAG |
| EGFP-Tat (37-48)-R | AATTCTTAGCCATAGGAGATGCCTAAGGCTTTTGTTATGAAACAAGC |
| Tat (37-48)-EGFP-F | TCGAGATGTGTTTCATAACAAAAGCCTTAGGCATCTCCTATGGCAG |
| Tat (37-48)-EGFP-R | AATTCTGCCATAGGAGATGCCTAAGGCTTTTGTTATGAAACACATC |
| RelB-F | CCCGAATTCATGCTTCGGTCTGGG |
| RelB127-F | CCCGAATTCATGCTGGTCATCACGGAG |
| RelB236-F | CCCGAATTCATGGAGGCTGCCATTGAG |
| RelB378-F | CCCGAATTCATGAACGTCTTCCTGCAGC |
| RelB285-R | TTACTCGAGTTAATCCATCCGGCGCATCTG |
| RelB377-R | TTACTCGAGTTAGACTGTCACGGGCTCGAC |
| RelB579-R | TTTCTCGAGTTACGTGGCTTCAGGCCC |
| RelB299-R | CCCCTCGAGTTAGTTTGTGGATTTCTTGTC |
| LTR-46-F | AAAGGTACCGAGCCCTCAGATGCTGC |
| LTR-83-F | TTTGGTACCTCCAGGGAGGCGTGG |
| LTR-120-F | ATAGGTACCATCGAGCTTGCTACAAGGGAC |
| LTR-160-F | ATAGGTACCCCCGAGAGCTGCATCCG |
| LTR-218-F | TACGGTACCCCCTGAGAGAGAAGTGTTAG |
| LTR-R | CCCCTCGAGCAAGCTTTATTGAGGC |
| mutNF-κB-F | GAGCTTGCTACAATCTACTTTCCGCTGTCTACTTTCCAGGGAG |
| mutNF-κB-R | CTCCCTGGAAAGTAGACAGCGGAAAGTAGATTGTAGCAAGCTC |
| mutTAR-F | CTCTGGTTAGACCAGACCCGAGCCTGGGAGCTC |
| mutTAR-R | GAGCTCCCAGGCTCGGGTCTGGTCTAACCAGAG |

Table 2 List of siRNAs used in RNAi.

| Name | Sequence (5’-3’) |
| --- | --- |
| NC-sense | UUCUCCGAACGUGUCACGUTT |
| NC-antisense | ACGUGACACGUUCGGAGAATT |
| RELB1-sense | GGAAGAUUCAACUGGGCAUTT |
| RELB1-antisense | AUGCCCAGUUGAAUCUUCCTT |
| RELB2-sense | CCGUGACAGUCAACGUCUUTT |
| RELB2-antisense | AAGACGUUGACUGUCACGGTT |
| RELB3-sense | CCAGGAGCACAGAUGAAUUTT |
| RELB3-antisense | AAUUCAUCUGUGCUCCUGGTT |
