# Supplementary figures and images for: Cellular RelB interacts with the transactivator Tat and enhance HIV-1 expression
Source: Retrovirology. 2018 Sep 21;15:65. doi: 10.1186/s12977-018-0447-9 (PMC6150996; doi:10.1186/s12977-018-0447-9)

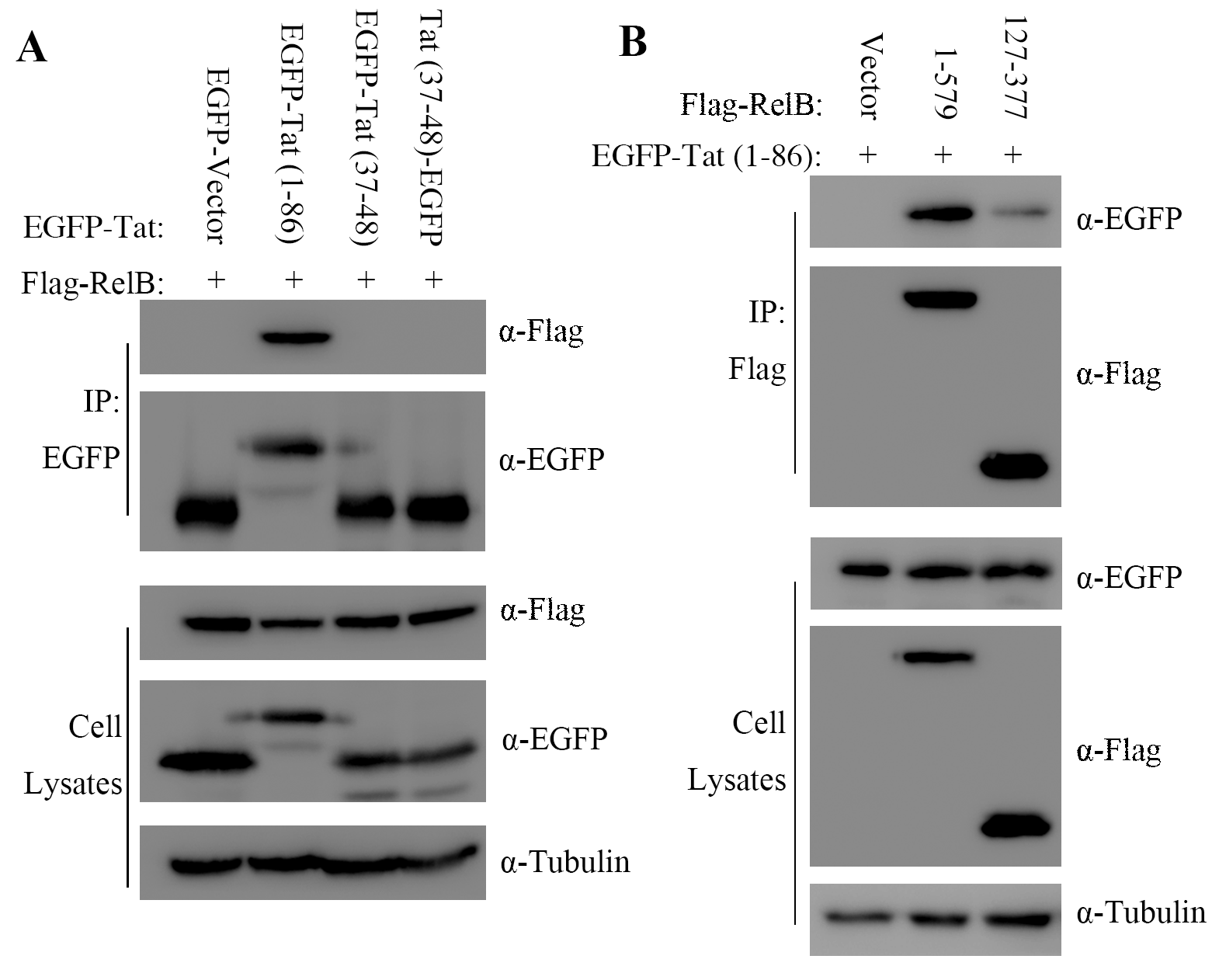

Supplement: Supplementary file 2 — Additional file 2. Identification of Tat and RelB interaction minimal domains. (A) pFlag-RelB (3 μg) was transiently transfected into HEK 293T cells (4 × 10^6^) together with the wild type Tat or truncation mutants (3 μg). Tat proteins were immunoprecipitated (IP) (anti-EGFP), and immunoblotted with the indicated antibodies. (B) HEK 293T cells (4 × 10^6^) were transfected with pEGFP-Tat (3 μg) and the indicated Flag-tagged deletion mutants of RelB (3 μg). RelB was immunoprecipitated (IP) (anti-Flag), and immunoblotted with the indicated antibodies. [file 12977_2018_447_MOESM2_ESM.tif]

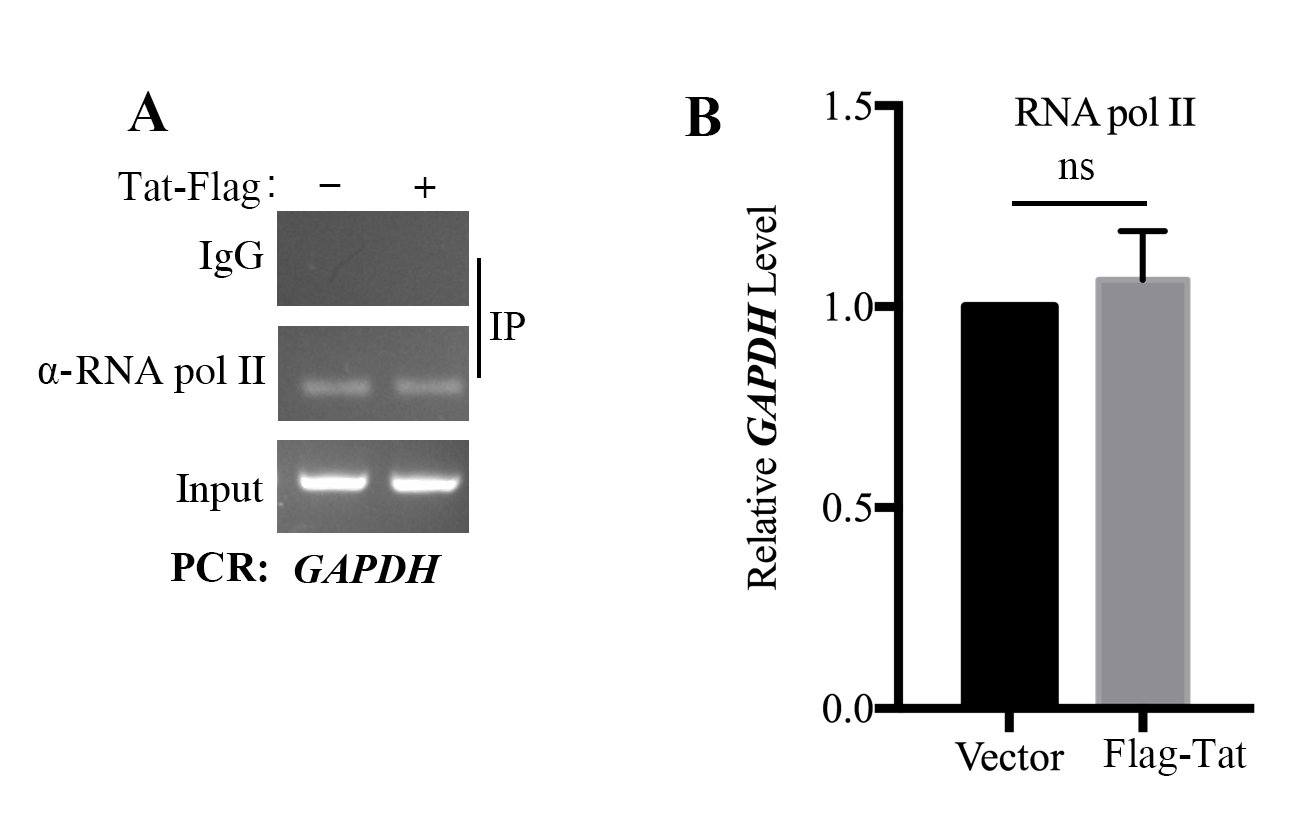

Supplement: Supplementary file 3 — Additional file 3. Immunoprecipitation of GAPDH-associated RNA Pol II was not Tat dependent. (A) TZM-bl cells were transfected with pFlag-Tat (6 μg) or vector plasmids. Fixed chromatin in these cells was immunoprecipitated with the anti-RNA Pol II antibodies. Samples were assessed for enrichment in transcriptionally active control DNA (GAPDH) by UV visualization of PCR products in an ethidium bromide-stained agarose gel. (B) Relative levels were quantified by real-time PCR analysis performed in triplicate and normalized to input controls. [file 12977_2018_447_MOESM3_ESM.tiff]

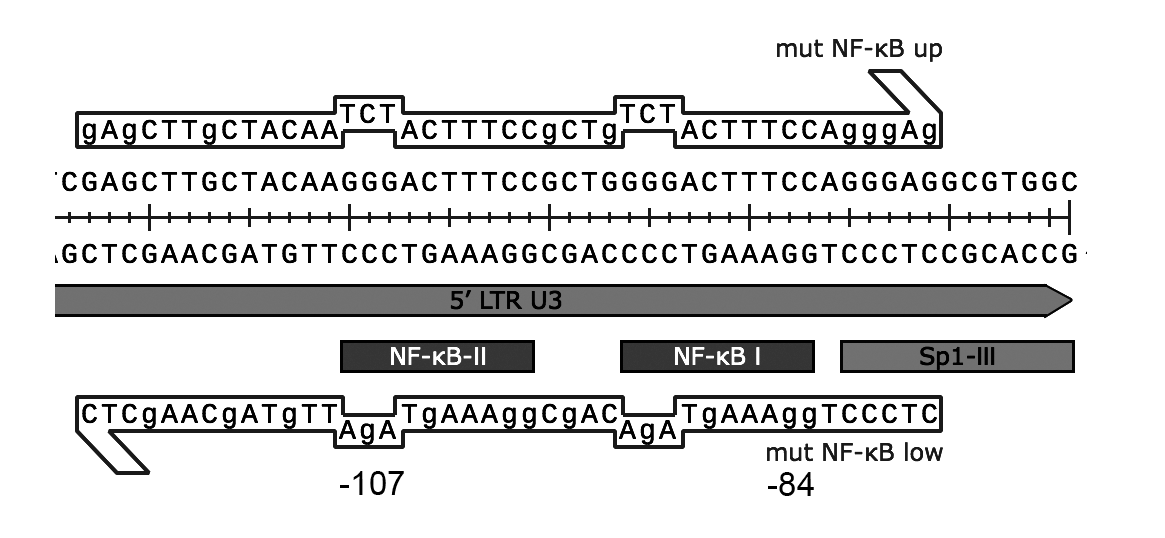

Supplement: Supplementary file 4 — Additional file 4. Model of mutNFκB reporter plasmids. NF-κB mutation of the HIV LTR reporter plasmids were generated using PCR-based mutagenesis. [file 12977_2018_447_MOESM4_ESM.tiff]

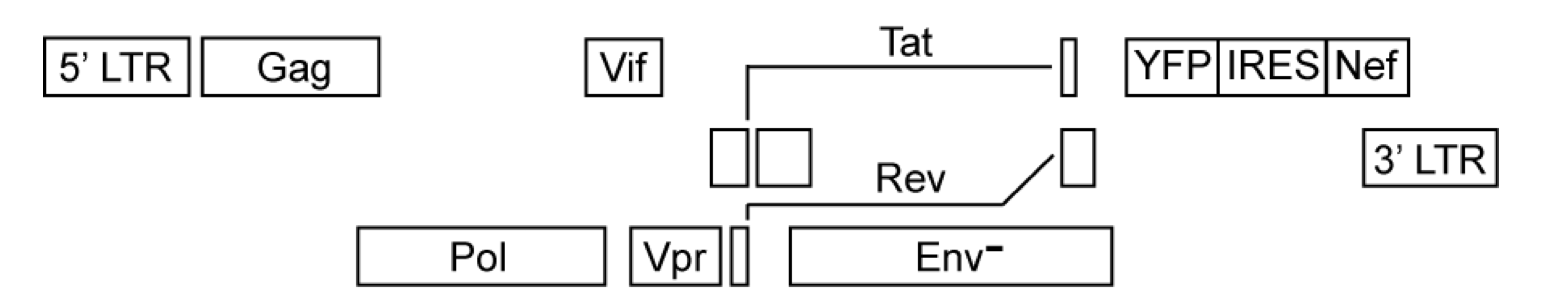

Supplement: Supplementary file 5 — Additional file 5. Schematic presentation of the NLENY1-ES-IRES proviral DNA. [file 12977_2018_447_MOESM5_ESM.tif]
